# Ear Dyskinesia in the Absence of Neuroleptics: A Case Report

**DOI:** 10.7759/cureus.63637

**Published:** 2024-07-01

**Authors:** Mohammed Azzahrani, Abeer Algasim

**Affiliations:** 1 Family Medicine, Rosedale Medical Center, Toronto, CAN

**Keywords:** moving ear syndrome, external ear movement, case report, botulinum toxin (botox) treatment, neuroleptics, involuntary movements, ear dyskinesia

## Abstract

Ear dyskinesia, also known as "moving ear syndrome," is a rare movement disorder characterized by involuntary, rhythmic, or semi-rhythmic contractions of the external ear muscles. The condition is not well-documented in the medical literature, with only a few case reports available. We present the case of a 37-year-old teacher from Saudi Arabia who developed a history of sudden, progressive involuntary movement of the posterior head region, provoking movement of the external ears, over the course of one year. The movements were non-rhythmical, more prominent on the right side, and associated with occasional involvement of the face and anterior neck muscles. The patient had no history of neuroleptic use or other relevant medical conditions. Examination confirmed the presence of palpable muscle contractions originating mainly from the posterior region, with the movements not synchronized across the two sides. Investigations, including blood tests and brain MRI, did not reveal any underlying pathology. A diagnosis of ear dyskinesia was made, and botulinum toxin treatment was recommended; however, the treatment showed no results, and then the patient was subsequently lost to follow-up. This case adds to the limited literature on the rare phenomenon of ear dyskinesia, highlighting the clinical presentation and the challenges in the management of this unusual movement disorder. Further research is needed to better understand the underlying mechanisms and optimal treatment approaches for this condition.

## Introduction

Dyskinesia is a movement disorder characterized by involuntary, erratic, and often repetitive movements [1,2]. These movements can range in severity from mild to severe, affecting the face, arms, legs, and trunk among other regions of the body [3]. Although the exact cause of ear dyskinesia is unknown, neuroactive medications like methylphenidate, selective serotonin reuptake inhibitors (SSRIs), and neuroleptics have been commonly associated with the condition [4]. Long-term use of neuroleptics, generally referred to as antipsychotic medications, is one of the most common causes of dyskinesia [5-7]. Additionally brought on by blepharospasm, oromandibular dystonia, and Meige syndrome [2].

Ear dyskinesia, or localized involuntary movements of the ear, has not been well investigated. According to Dr. Harsha Singh, the term "moving ear syndrome (MES)" which refers to movement abnormalities involving the ear, is not often mentioned in the literature. These disorders include auricular myoclonus, focal motor seizures, and dystonia [8]. The anterior, superior, and posterior auricular muscles are the three vestiges around the ear [9]. Extrinsic scalp muscles can be used by certain people to move their ears, despite the intrinsic musculature of the ear being involuntary [10].

According to Dr. V. Yahya et al., the rhythmic or semi-rhythmic contractions of the external ear muscles in moving ear syndrome (MES) can be painful or uncomfortable, but these movements typically stop during sleep or when the person voluntarily moves their face. Pharmaceutical options such as propranolol, clonazepam, and pregabalin are used as treatment approaches; however, injections of botulinum toxin have been found to be the most successful treatment [2,11,12]. Pallidothalamic tractotomy has also been used anecdotally [4,9]. The motions' clinical and electrophysiological features bear similarity to dystonic patterns of muscle contraction [13]. Restricted and isolated dystonic movements of cranial musculature, such as the muscles of the pinna, are exceedingly rare, despite the fact that segmental dystonia of the cranial and upper limb muscles is widely recognized [12]. Due to its rarity, the medical literature on ear dyskinesia is limited, with only a handful of case reports and studies examining this phenomenon.

## Case presentation

A 37-year-old male teacher from Saudi Arabia is a non-smoker and non-alcoholic, presented with a year history of a warm feeling in his palms and sudden, progressive, involuntary movements of the posterior head region. These movements caused his external ears to move (Video 1). Occasionally, he also noticed involvement of his face and the area under his eyes, along with twitch-like movements in the anterior neck muscle region. He did not report any clear exacerbating or relieving factors, nor have any headaches, blurred vision, or previous similar history or serious illnesses. The patient had no history of neuroleptic use or any other medications, except for Vitamin D3 50,000 IU per day.

**Video 1:** Ear dyskinesia

On examination, the patient exhibited repeated, non-rhythmical, significant amplitude movements of his external ears, which appeared more frequent and vigorous on the right side compared to the left, although the degree of asymmetry was not remarkable. Palpable muscle contractions, originating mainly from the posterior region, were present. Both sides were simultaneously involved, but the movements were not synchronized across the two sides. His gait, power, reflexes, and other neurological exams were normal. There was no evidence of neck dystonia or any other unusual features of the head or neck. Ophthalmic examination, including the optic fundi, was unremarkable, with no nystagmus, motor deficits, or cerebellar or sensory abnormalities noted.

Laboratory tests, including blood work and thyroid-stimulating hormone (TSH) levels, were within normal limits. The patient was non-immune to hepatitis B. Brain MRI did not reveal any intracranial abnormalities, but a mucus retention cyst in the left maxillary sinus suggested chronic inflammatory changes.

The diagnosis was ear dyskinesia. The Botox treatment was injected, but the patient did not respond to it. The patient was scheduled for a follow-up appointment, but he returned to Saudi Arabia before the appointment.

The patient provided informed consent for all diagnostic and therapeutic procedures, examinations, and to document the case with a video.

## Discussion

In the presented case, ear dyskinesia has been present for one year without the use of neuroleptics, local trauma, or surgery in an otherwise healthy young male. There have not been many case reports or literature reviews on ear dyskinesia; Carluer L et al. reported a 57-year-old woman who complained of involuntary movements of both of her ears for the past year. The movements were initially intermittent but gradually became continuous and she was unable to control them voluntarily. The movements disappeared when she was sleeping. There was no history of local trauma or prior treatment with neuroleptic medications, but the patient had started taking paroxetine for depression three months prior to the onset of the ear movements. On examination, there were semi-rhythmic, synchronous movements of elevation and retraction of both ears, as well as visible contractions in the skin around the ears. There were also intermittent contractions of the frontal muscles. The patient showed signs of amimia (lack of facial expression) and slow alternating serial movements, but no tremor or hypertonia. A brain MRI shows no alteration. Electromyography (EMG) of the left auricularis superior muscle showed normal motor unit potentials occurring in bursts at a frequency of 2 Hz. The patient was treated with local injections of 40 units of botulinum toxin type A (Botox) into the auricularis superior and posterior muscles, without EMG guidance. The treatment led to positive results, significantly decreasing the frequency and intensity of the involuntary ear movements [2].

Also, Singh H et al. reported a 17-year-old female student who presented with complaints of headache and involuntary movement of both external ears [8]. The headache had started about a year prior to the ear movements, and various medications including amitriptyline, escitalopram, mirtazapine, clonazepam, and carbamazepine were tried unsuccessfully to treat the headache. After six months, the patient started to notice bilateral, semi-rhythmic, involuntary movements of her external ears, with elevation and retraction at a frequency of 30-40 movements per minute. These movements were absent during sleep and decreased slightly with distraction but were not associated with any other abnormal movements or symptoms. The patient's medical history was otherwise unremarkable, and neurological workup including blood tests, MRI, and EEG was all normal. A CT scan showed bilateral sinusitis. After simplifying the patient's medication regimen, the headache improved but the ear movements persisted. Tetrabenazine was then started and titrated up to 75 mg/day, which resulted in a significant reduction in the frequency and amplitude of the ear movements [8].

Another case involved a 55-year-old man who presented with involuntary, repetitive backward twitching of the right ear, associated with discomfort and inner tension. The movements could not be suppressed and persisted during sleep. The patient had a history of severe traumatic brain injury with left temporal encephalomalacia, followed by involuntary left ear wiggling that partially resolved with transitory administration of phenobarbital. Pharmacological treatment with clonazepam was ineffective for the right ear dyskinesia. Needle EMG revealed rhythmic bursts from the right auricularis posterior muscle. The patient underwent EMG and ultrasound-guided injection of 10 units of botulinum toxin A into the affected muscle, which resulted in a significant reduction in the frequency of the twitching and complete disappearance during sleep [4].

Finally, a study included two patients. Patient 1 was a 23-year-old white man who developed continuous, semi-rhythmic contractions of variable amplitude involving both ears and scalp muscles, with more pronounced involvement on the right side and experienced right temporal pain and a fluttering noise in the left ear. Patient 2 was a 32-year-old right-handed man of West Indian extraction who had a 12-year history of involuntary movement of the left ear and had been treated for schizophrenia with neuroleptic medications. Both patients exhibited slow, rhythmic movements of the ear, with a superimposed jerky element, suggestive of focal dystonia with myoclonic jerks. Investigations, including brain MRI, were unremarkable in both cases, and the patients responded well to treatments for focal dystonia, such as clonazepam for patient 1 and botulinum toxin injections for the patient [11].

Similar to the case reports, our patient experienced involuntary movements of the ear with no associated trauma or neuroleptic use. Likewise, the progressive nature of the patient’s dyskinesia to surrounding regions was also seen in published case reports where the patient exhibited a pulling sensation associated with her ear and left neck that occasionally spread to the face and shoulder. Reporting this rare case of ear dyskinesia is significant as it adds to the limited body of medical literature on this uncommon movement disorder. Sharing the findings and management strategies can enhance understanding and awareness among clinicians, potentially guiding diagnosis and treatment for future cases of ear dyskinesia.

## Conclusions

Ear dyskinesia is an uncommon medical condition with few published studies and reports exploring its causes and treatment. It is supposed to be associated with the use of neuroleptics; however, none of the presented case reports support this conclusion. Botox seemed a suggestive treatment for ear dyskinesia; however, its efficacy is supported by few reports. Therefore, alternative therapeutic strategies are in need of more research. Further research is required to rule out neurological causes and determine the best-suited treatment for ear dyskinesia. Additionally, tetrabenazine and other VMAT2 inhibitors have emerged as effective treatments for various forms of dyskinesia and may warrant investigation for managing ear dyskinesia as well. 
